# Clinical Management of Dewey’s Type 3 Anterior Crossbite in Angle Class I Malocclusion: A Case Report

**DOI:** 10.7759/cureus.67568

**Published:** 2024-08-23

**Authors:** Rushikesh M Kachave, Kushal P Taori, Vikrant V Jadhav, Priyanka Paul, Prem A Sawarbandhe, Rohan R Khetan

**Affiliations:** 1 Orthodontics and Dentofacial Orthopedics, Sharad Pawar Dental College and Hospital, Datta Meghe Institute of Higher Education & Research, Wardha, IND; 2 Public Health Dentistry, Sharad Pawar Dental College and Hospital, Datta Meghe Institute of Higher Education & Research, Wardha, IND

**Keywords:** temporomandibular, occlusal, proclination, crossbite, catalan

## Abstract

Anterior crossbite is a significant malocclusion characterized by the lingual eruption of one or more maxillary front teeth, often impeded by the mandibular deciduous counterparts. This clinical case study focused on a patient with an anterior crossbite involving the central and lateral incisors, detailing the diagnosis, treatment course, and outcomes. During the clinical evaluation, both aesthetic concerns related to attrition and occlusal malocclusion were identified. Diagnostic tests, including radiographs and extraoral and intraoral examinations, were conducted before initiating treatment. The treatment aimed to correct the anterior crossbite while enhancing function and aesthetics. Fixed orthodontic appliances were introduced to facilitate precise tooth movement. Throughout the first year, the alignment, crossbite correction, and occlusal refinement were meticulously documented through photographs. The treatment resulted in improved occlusal relationships, functional bite, and enhanced smile aesthetics. This case report underscores the importance of comprehensive and functional orthodontic treatment in effectively resolving anterior crossbite and providing optimal patient care.

## Introduction

Dental malocclusion is marked by an irregular arrangement of the teeth, with the primary dentition being a key determinant. Anterior crossbite, an imbalance on the sagittal plane, causes the mandible to shift forward [1]. This condition can present as an underbite, an overbite, or a combination of both in skeletal components, with the prognosis often influenced by inherited factors. Anterior dental crossbite typically results from incisor inclination defects, such as lingualized occlusion of the upper incisors or flare of the lower incisors. An anterior skeletal or functional crossbite during primary dentition can lead to a terminal mesial plane in the deciduous second molars [2]. Insufficient space may cause permanent tooth buds to emerge in a crossbite, positioned palatally to the arch’s line, with the permanent tooth buds developing lingually to their deciduous counterparts [3]. This condition often results in permanent dentoalveolar crossbites in the anterior region of the jaws.

The Lower Inclined Bite Plane, or Catlan’s device, utilizes Newton’s third law of motion. The resin slope labially tips the front teeth while gently tipping the mandibular tooth lingually. This approach offers a quick, simple, affordable, and safe method for treating crossbites, avoiding the need for fixed orthodontic tooth movement procedures, making it a cost-effective option [4].

If left untreated, an anterior crossbite can lead to various complications, including attrition, gingival recession, alveolar bone loss, temporomandibular dysfunction, lower incisor mobility, and detrimental growth effects on the teeth, alveolar processes, and the skeletal components of the jaw and maxilla in the anterior region [5].

The temporomandibular joint (TMJ) is a compound articulation formed by the temporal bone and the mandibular condyle, with both surfaces covered by dense articular fibrocartilage. The temporal bone articulates with each cyst through the articular fossa, articular eminence, and preglenoid plane, distinguishing the TMJ from other joints due to the anterior translation of the condyle along the articular eminence [6]. Temporomandibular dysfunction is a common condition in dental practice, with osteoarthritis - a degenerative change marked by cartilage deterioration - being a significant concern. This deterioration is characterized by a decrease in chondrocyte count and alterations in the extracellular matrix [7].

The diagnosis and cause of crossbite guide the treatment approach. While dentoalveolar movement can correct tooth and alveolar disharmonies, significant skeletal imbalances may necessitate surgical intervention [8]. The primary goals of treatment are to improve occlusion, enhance the aesthetics of the dentofacial complex, and prevent complications such as enamel abrasions, anterior tooth fractures, and periodontal pathosis [9].

## Case presentation

A 22-year-old male patient was reported to the outpatient department of Sharad Pawar Dental College and Hospital, Wardha, and was referred to the Department of Orthodontics and Dentofacial Orthopedics with a chief complaint of irregularly placed upper front teeth. Clinical examination revealed a mesocephalic head form, a mesoprosopic facial form with an obtuse nasolabial angle, and a shallow mentolabial sulcus. The clinical Frankfort mandibular angle was average, indicating an average growth pattern. There were no signs or symptoms of TMJ dysfunction, such as clicking, crepitation, pain, or deviation in mandibular closure.

**Figure 1:**
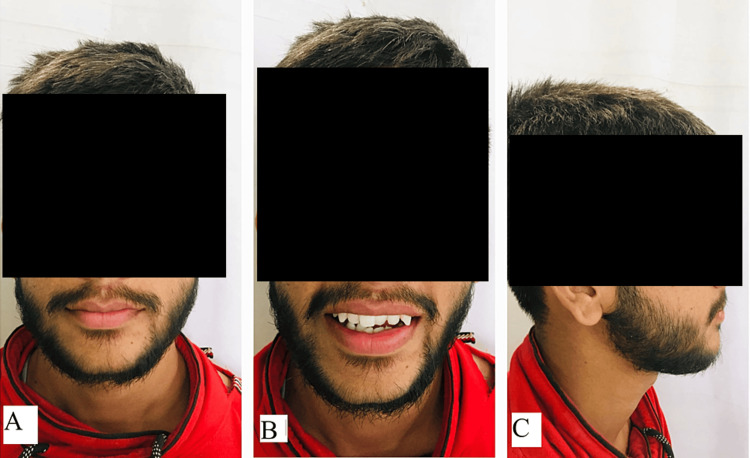
Pretreatment extraoral photographs: (A) frontal view, (B) smiling view, and (C) profile view

**Figure 2:**
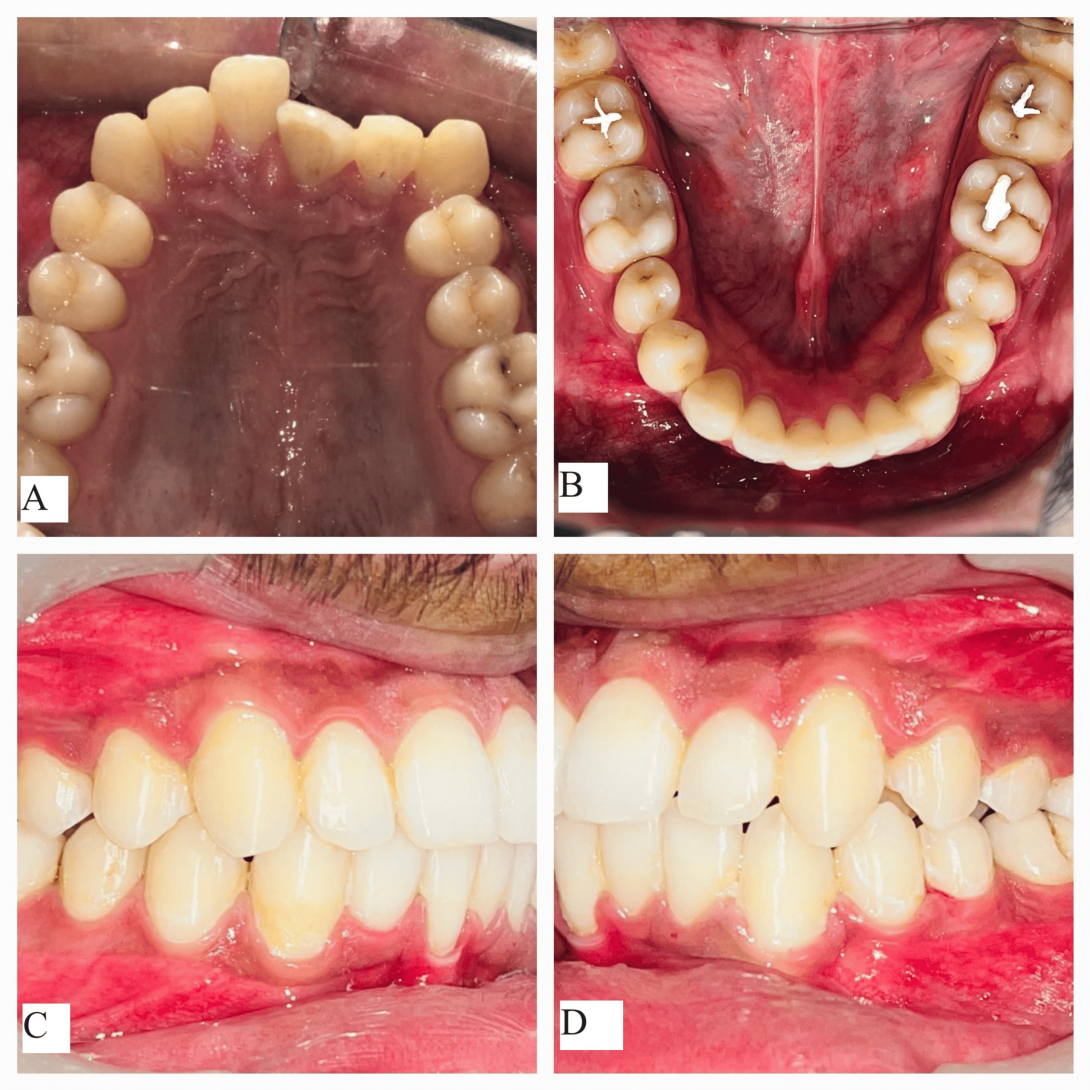
Pretreatment intraoral photographs: (A) maxillary arch, (B) mandibular arch, (C) right occlusion, and (D) left occlusion

**Figure 3:**
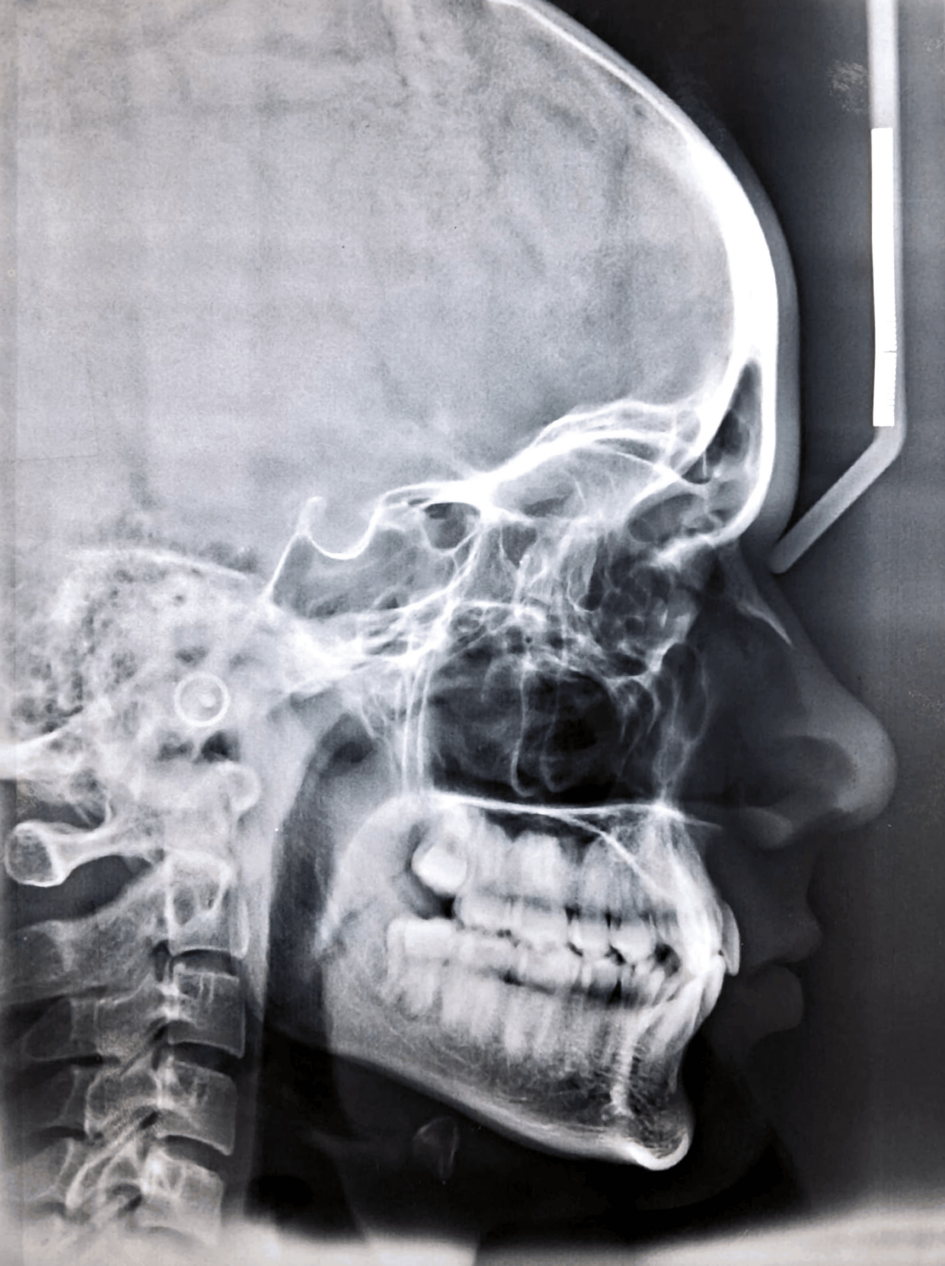
Pretreatment lateral cephalogram illustrating a Class I skeletal pattern

Intraoral examination showed lingually placed maxillary lateral incisors and a buccally placed maxillary left canine, indicating an anterior crossbite with teeth 21 and 22, as well as a crossbite with 31. Mild anterior crowding, proclined lower incisors, and rotated tooth 33 were noted in the lower jaw. Additionally, there were lingually tilted lower posterior teeth bilaterally and asymptomatic carious lesions on 36, 37, and 47. The patient’s bite revealed molars and canines in Angle Class I malocclusion. Radiographic evaluation showed no obstacles in the upper anterior region, and TMJ spacing was normal. Lateral cephalometric analysis revealed a decreased SNA angle due to retroclined upper anterior and an increased lower incisor to mandibular plane angle, indicating proclined lower incisors. Radiographically, there was a mild tendency toward Class III malocclusion. Overall, the diagnosis was Angle Class I malocclusion with Dewey’s modification Type 3 (anterior crossbite). Figure 1 shows pretreatment extraoral photographs; Figure 2 shows pretreatment intraoral photographs; Figure 3 shows cephalometric examination; and Table 1 and Table 2 present pretreatment and posttreatment comparisons using Tweed’s and Steiner’s analyses.

**Table 1:** Comparison of cephalometric measurements pretreatment and posttreatment (Tweed’s analysis) FMIA, Frankfort mandibular incisor angle; FMPA, Frankfort mandibular plane angle; IMPA, incisor mandibular plane angle

Parameters	Normal	Pretreatment	Posttreatment
FMPA	25	24	25
IMPA	90	93	90
FMIA	65	63	65

**Table 2:** Comparison of cephalometric measurements pretreatment and posttreatment (Steiner’s analysis)

Parameters	Normal	Pretreatment	Posttreatment
SNA	82	77	81
SNB	80	81	80
SND	76	76	76
Mandibular plane	32	29	30
Occlusal plane angle	14	11	13
Up. 1 to NA	22	19	23
Up. 1 to NA (mm)	4 mm	3 mm	4
Lo. 1 to NB	25	27	25
Lo. 1 to NB (mm)	4 mm	6 mm	4
Interincisal angle	131	134	130

Treatment objectives

**Figure 4:**
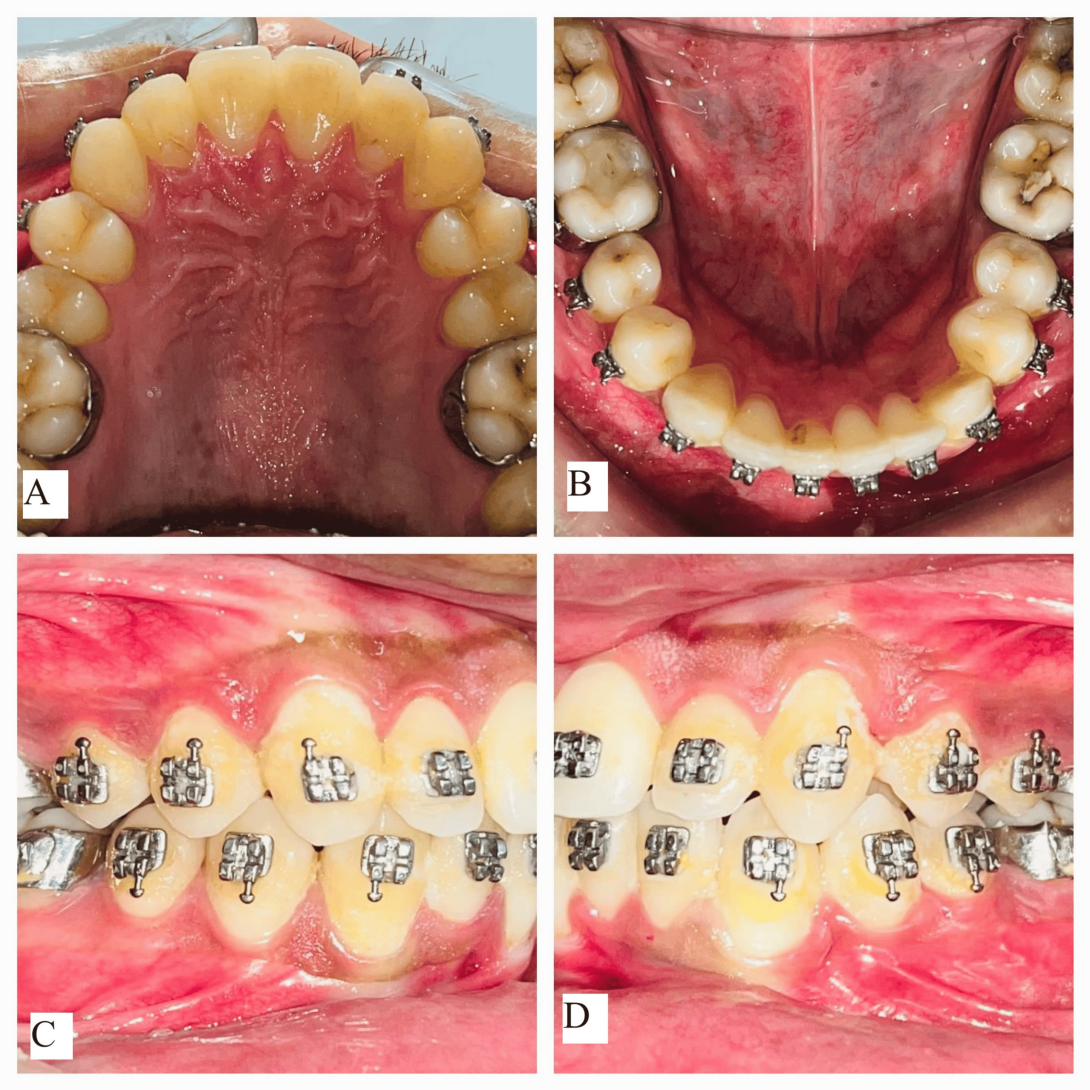
Mid-treatment intraoral photographs: (A) maxillary arch, (B) mandibular arch, (C) right occlusion, and (D) left occlusion

The treatment objectives were as follows: correction of the lingually placed upper anterior teeth and their inclination with respect to the basal bone; correction of the anterior crossbite; alignment of the lower anterior teeth; addressing proclination relative to the lower basal bone; and de-rotation of tooth 33. Additionally, the treatment aimed to improve the soft tissue profile and restore the carious lesions on teeth 36, 37, and 47. Figure 4 shows mid-treatment intraoral photographs.

Treatment plan progress

Bonding of the upper arch was completed first, using McLaughlin, Bennett, and Trevisi (MBT) brackets with a 0.022 slot dimension. Stainless steel brackets were bonded to the upper arch. To address the anterior crossbite, a bite elevation of 3 mm was performed on the lower molars. Initially, a 0.016-round NiTi wire was placed, followed by a 0.018-round NiTi wire to correct the upper anterior inclination (tip). Leveling and alignment were achieved as wire progression continued, culminating in the use of a 0.019 × 0.025 inch rectangular SS wire for torque correction.

The lower arch was bonded to address lower anterior crowding and rotation. Interproximal stripping of 3 mm was performed in the lower anterior region to settle the crowded teeth. A couple of forces were applied to tooth 33 for rotation correction; a couple of forces are defined as equal and opposite forces of the same magnitude along the same collinear path. After leveling and alignment with the 0.019 × 0.025 inch SS rectangular wire, occlusal settling was facilitated using vertical elastics on the buccal aspect.

Finishing and detailing were completed with artistic bands (second-order bends) on 0.017 × 0.025-inch rectangular Titanium Molybdenum Alloy (TMA) wires. TMA was chosen for its superior resiliency and stiffness, combining the properties of both SS and NiTi.

Treatment outcomes

**Figure 5:**
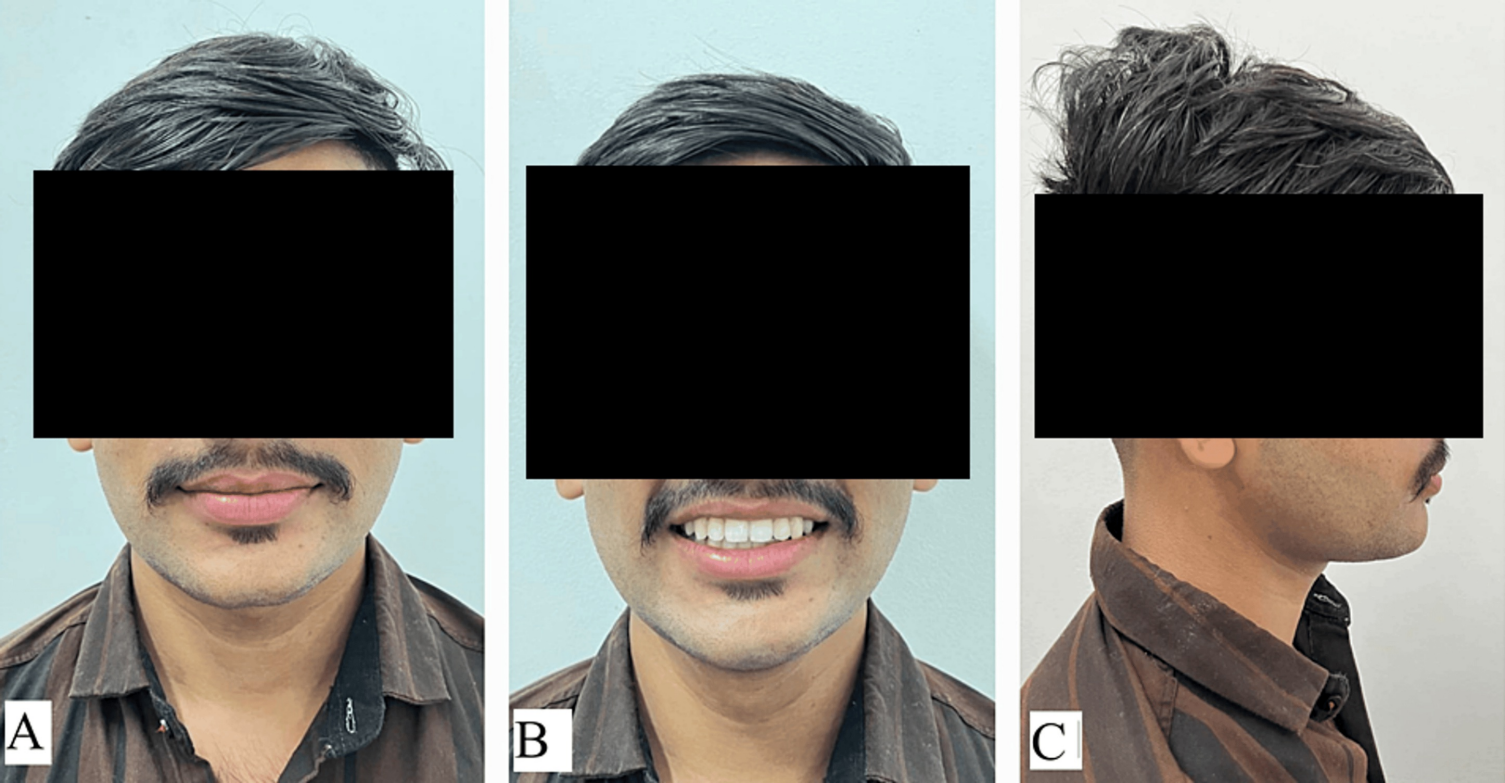
Posttreatment extraoral photographs: (A) frontal view, (B) smiling view, and (C) profile view

The treatment successfully corrected the lingually placed upper anterior teeth and their inclination with respect to the basal bone, as well as the anterior crossbite. Lower anterior crowding and proclination were relieved, and tooth 33 was de-rotated. The soft tissue profile was improved, and restorations were completed for teeth 36, 37, and 45. Figure 5 shows the posttreatment extraoral photographs of the patient.

## Discussion

Adult patients with anterior crossbite face unique challenges that require a comprehensive approach for successful repair and long-term stability. This discussion examines the management of anterior crossbite in a 22-year-old male patient, highlighting the difficulties encountered, patient concerns, and broader implications. Fixed orthodontic treatment with braces was the primary approach, providing precise control over tooth movement. For effective management, it is crucial to slow the movement of the upper front teeth to achieve proper interocclusion with the lower teeth, ensuring a correct bite. The primary challenge in adult anterior crossbite treatment is achieving optimal tooth movement while avoiding periodontal disease or root resorption [10].

The patient’s cooperation ensured successful orthodontic procedures, periodic examinations, and oral hygiene. Initial discomfort and pain, particularly following adjustments, were managed with analgesics and orthodontic wax, which effectively alleviated irritation and soreness [11].

Proper correction of the anterior crossbite reduces the risk of dental issues such as wear on teeth and TMJ disorders, restores proper occlusal function, and improves aesthetics. Posttreatment retention procedures were implemented to prevent recurrence and maintain alignment. Ongoing stability and timely responses to new issues through regular follow-up appointments are crucial for the long-term success of orthodontic therapy [12].

## Conclusions

Treating anterior crossbite necessitates a thorough approach that addresses functional and aesthetic concerns. In this case, an individualized treatment plan tailored to the patient’s specific needs was crucial. Successful outcomes depended on patient compliance and effective collaboration between the orthodontist and the patient. Fixed appliances, particularly braces, enabled precise control over tooth movement, allowing for gradual and accurate correction of the upper front teeth. This method enhanced both aesthetic results and occlusal function while preventing potential dental wear and TMJ issues related to occlusion disorders.

The treatment also emphasized managing patient discomfort and ensuring adherence to the plan. Initial discomfort was effectively controlled with symptomatic relief and open communication between the patient and the orthodontist. This supportive approach facilitated a smoother treatment experience, ultimately leading to improved oral health and long-term stability.
